# Quantitative Evaluation of Focus Measure Operators in Optical Microscopy

**DOI:** 10.3390/s25103144

**Published:** 2025-05-16

**Authors:** Weiying Piao, Yongqi Han, Liye Hu, Chunxue Wang

**Affiliations:** The Higher Educational Key Laboratory for Measuring & Control Technology and Instrumentation of Heilongjiang Province, Harbin University of Science and Technology, Harbin 150080, China; 2220610178@stu.hrbust.edu.cn (Y.H.); 2220610117@stu.hrbust.edu.cn (L.H.); 2320610115@stu.hrbust.edu.cn (C.W.)

**Keywords:** automatic focusing, optical microscopy, focus measure operator, evaluation metrics

## Abstract

Given the variety of focus measure operators, selecting an appropriate one based on scene requirements is critical. This study designs evaluation metrics based on the morphological characteristics of focus measure curves. A multipoint linear fitting method is proposed to partition the curve into steep slope and gradual slope regions, from which four metrics are derived: the steep slope region width (*W_s_*), the steep to gradual ratio (*R_sg_*), the curvature of peak point (*C_p_*), and the relative root mean square error (*RRMSE*). Several representative focus measure operators were chosen for experimental evaluation. The results demonstrate that when image acquisition parameters or image content change, the performance evaluations of most operators remain stable. Therefore, the proposed metrics robustly characterize the performance and features of various operators. These metrics serve as a valuable reference for selecting appropriate operators in optical microscopy and provide a theoretical foundation for designing new ones.

## 1. Introduction

Focus measure operators (FMOs), also referred to as focusing functions, are essential tools for assessing image sharpness. They are widely used in autofocus systems and 3D surface reconstruction [1,2,3,4,5]. Given the extensive variety of FMOs, selecting the most suitable one based on scene characteristics and system requirements remains a challenging task.

The selection of FMOs requires a comprehensive consideration of factors such as image content features, algorithm real-time performance, and sensitivity to image quality variations. To improve the overall performance of focus evaluation algorithms, researchers have not only improved classical FMOs [6,7,8,9,10], but have also continuously explored and proposed new evaluation methods to better meet the demands of specific applications [11,12,13,14,15]. However, the performance of different FMOs varies significantly across the same scene, while the same operator may exhibit different behaviors in varying scenarios. Therefore, objectively measuring their performance remains a topic worthy of in-depth study.

To systematically evaluate FMO performance, researchers have proposed numerous methods. Frans C.A. Groen et al. examined the performance of various focus functions for autofocus applications under different image types. They experimentally compared 11 focus evaluation functions and analyzed them based on metrics such as unimodality, accuracy, reproducibility, range, and general applicability [16]. Similarly, Sun et al. compared 18 focus evaluation algorithms for microscopic image processing, ranking their performance using criteria like precision, range, number of false maxima, width, and noise robustness [17]. Sun et al. further analyzed the computational principles of different FMOs to assess their suitability for autofocus, considering factors such as computational speed, uniqueness, accuracy, and sensitivity [18]. Zhai et al. designed six quantitative evaluation metrics—steep region width, sharpness ratio, steepness, flat region fluctuation, local extremum factor, and sensitivity—based on focus curve characteristics. Using these metrics, they quantitatively assessed 12 typical FMOs and provided recommendations for optimal selection [19]. To investigate the role of FMOs in 3D surface reconstruction, Said Pertuz et al. explored their application in depth recovery, combining them with depth information to reconstruct 3D depth maps and evaluating the results using root mean square error (RMSE) [20].

Although existing methods have partially revealed performance differences among focus measure operators, they remain insufficient for objective and accurate performance evaluation. The focusing curves vary with imaging scenes, which compromises the stability of evaluation results. For instance, the unimodality characteristic is intrinsically related to the measured object; the focusing curve exhibits unimodality only when the object approximates a planar surface [19]. The accuracy metric relies on prior knowledge of the “truth focus” position, which is often unavailable in practical applications. In complex scenes, subjective determination or labeling errors of focus positions directly affect the accuracy metric, thereby undermining its reliability. The steep/flat region partitioning method proposed in [19] requires threshold selection that is content-dependent. Inappropriate threshold choices may lead to erroneous region segmentation, rendering the evaluation metrics invalid.

In autofocus systems, test images are formed at different distances. The FMO is applied to each frame to quantify image sharpness. The focus measure values plotted against longitudinal position (frame number) form the focus measure curve (also known as the focusing curve). Different FMOs exhibit distinct focusing curve shapes; therefore, the morphological characteristics of focusing curves can effectively reflect the performance of FMOs. To address the limitations of current evaluation systems in terms of objectivity and accuracy, this paper develops a set of quantitative evaluation metrics based on the morphological characteristics of focus measure curves. The proposed metrics comprehensively consider adaptability to focusing curve variations, enabling the thorough characterization of the FMOs’ capabilities, including sensitivity and robustness.

## 2. Design of Quantitative Metrics

### 2.1. Selection of the Cutoff Point

**Figure 1:**
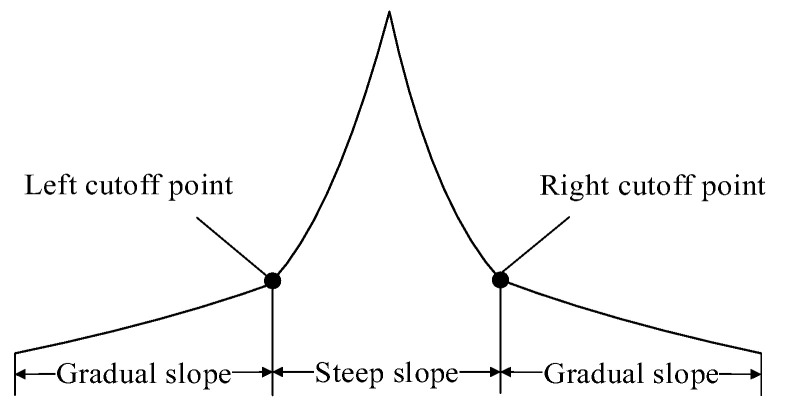
Diagram of the focusing curve.

The focusing function value changes rapidly near the focus, resulting in a steep curve, while it changes slowly away from the focus, leading to a relatively flat curve. Therefore, the focusing curve can be segmented into steep slope region near the focus and gradual- slope region away from the focus, as illustrated in Figure 1.

**Figure 2:**
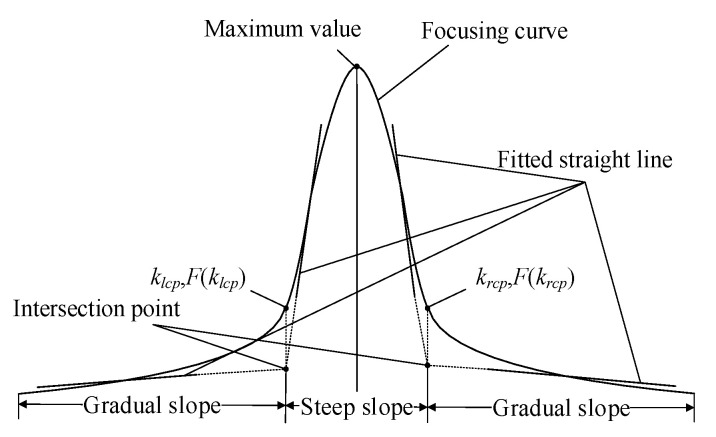
Diagram of the left and right cutoff points.

Determining the cutoff points between the steep slope and gradual slope regions is a critical issue. The focusing curve exhibits approximately linear sections within both regions. These linear sections can be fitted with straight lines, and their intersection point defines the horizontal coordinates of the cutoff points. Points on the focusing curve corresponding to these intersection coordinates are then identified as the cutoff points (Figure 2). The proposed method, based on multipoint linear fitting, demonstrates strong robustness and stability in experimental tests, enabling the objective and accurate segmentation of the focusing curve.

For the gradual slope regions, the fitting effect can be evaluated using the residual sum of squares (*RSS*) between the focusing curve and the fitted line, i.e., the least-squares regression line. For the steep slope region, the slope is much larger than that of the gradual slope regions, resulting in a significantly larger *RSS*. This poses challenges when using *RSS* as a metric in practical applications. In general, as the curve value increases, the slope of the fitted straight line typically increases initially and then decreases. There is a region where the fitted straight line has the maximum slope. The results demonstrate that the slope difference between the maximum slope fitted line and the least-squares fitted line in the steep regions is negligible. Therefore, the slope of the fitted straight line can serve as a discriminative basis for measuring the fitting accuracy of the steep slope region. The fitted straight line with the largest slope is considered the fitted straight line of the steep slope region.

The focusing curve may present instances where a certain number of points happen to be perfectly collinear. This phenomenon can occur in any region of the focusing curve, with its occurrence probability being inversely proportional to the number of points involved: the fewer the points, the higher the probability. A typical case would be three perfectly aligned points. In such scenarios, when constructing a fitting line using exactly these three points, the least-squares method will yield erroneous results. Consequently, when the number of points used for line construction is too small, this paper proposes using the slope as the discriminant criterion for line fitting in gradual slope regions, where the fitted line with the minimum slope should be identified as the optimal fitting line.

The images are indexed by *k*. The horizontal coordinates of the intersection points are denoted as *k_lcp_* and *k_rcp_*, and the focusing function values are denoted as *F*(*k_lcp_*) and *F*(*k_rcp_*). Due to the minimum step size limitation of the stepper motor, the focusing curve can only take discrete values. Since *k_lcp_* and *k_rcp_* are non-integer values, *F*(*k_lcp_*) and *F*(*k_rcp_*) cannot be directly obtained. The method of linear interpolation is used to obtain the values of *F*(*k_lcp_*) and *F*(*k_rcp_*).

### 2.2. Steep Slope Region Width (W_s_)

The steep slope region width (*W_s_*) is an important feature of the focusing curve. A narrow *W_s_* indicates high sensitivity to focus changes, while a wider width suggests lower sensitivity. The *W_s_* is defined in Equation (1).

(1) $$W_{s}=d\left(k_{rcp}-k_{lcp}\right)$$

In Equation (1), *d* represents the step distance of the image acquisition system. However, in certain cases, *d* may be unknown. Under such circumstances, *d* can be omitted to enable continued investigation of the operator’s performance.

### 2.3. Steep to Gradual Ratio (R_sg_)

The focusing curve is significantly different in the steep and gradual slope regions. Generally, the flatter the gradual slope region, the steeper the steep slope region. In order to comprehensively measure this morphological feature of the focusing curve, the steep to gentle ratio (*R_sg_*) is proposed, which is defined in Equation (2).

(2) $$R_{sg}=\frac{2F\left(k_{max}\right)-F(k_{lsm})-F(k_{rsm})}{F(k_{lmax})-F(k_{lmin})+F(k_{rmax})-F(k_{rmin})}$$

In Equation (2), *F*(*k_max_*) is the peak point of the focusing function. *F*(*k_l__sm_*) and *F*(*k_rsm_*) are the lowest points in the left and right steep slope regions, respectively. *F*(*k_lm__ax_*) and *F*(*k_rmax_*) are the highest points in the left and right gradual slope regions, while *F*(*k_lm__in_*) and *F*(*k_rmin_*) are the lowest points in those same gradual slope regions. The *R_sg_* serves as a sensitivity index: a higher value indicates a better ability to distinguish between clear and blurred images.

It should be noted that the number of collected images affects the *R_sg_* index. Therefore, this paper stipulates that the number of collected images should be more than twice the *W_s_*. Generally, as long as this requirement is met, increasing the number of collected images will not significantly change the *R_sg_* index.

### 2.4. Curvature at Peak (C_p_)

**Figure 3:**
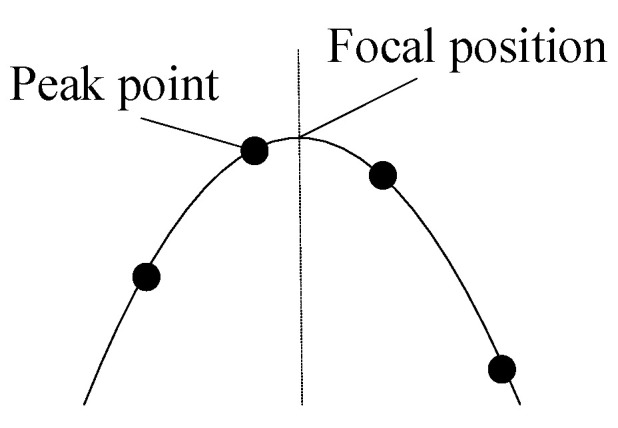
Peak point out of focal position.

For applications such as autofocus systems and shape from focus (SFF), the sensitivity of the focusing function to focal variations is crucial. The shape of the focusing curve directly reflects the operator’s ability to perceive focal deviations. Generally, a sharper focusing curve at its peak indicates greater sensitivity to focal deviations. The peak point of the focusing curve and the focal position generally do not coincide, as shown in Figure 3.

Since the peak point does not coincide with the focal position, methods that evaluate FMO sensitivity by calculating the height difference or slope between the peak and adjacent points are not accurate enough. Noting that the curvature of the focusing curve remains approximately constant near the peak, this paper proposes using the peak point curvature of the focusing curve to measure the sensitivity of the FMO at the focal position. This curvature metric denoted as *C_p_* is formally defined in Equation (3).

(3) $$C_{p}=\frac{\left|F^{\prime \prime }(k_{max})\right|}{{\left\{1+{\left[F^{\prime }(k_{max})\right]}^{2}\right\}}^{3/2}}$$

*F′*(*k_max_*) is the first derivative of the focusing curve at the peak point, as shown in Equation (4); *F″*(*k_max_*) is the second derivative of the focusing curve at the peak point, as shown in Equation (5).

(4) $$F^{\prime }(k_{max})=\frac{F(k_{max}+1)-F(k_{max}-1)}{2d}$$

(5) $$F^{\prime \prime }(k_{max})=\frac{1}{d}\left[F(k_{max}+1)-2F(k_{max})+F(k_{max}-1)\right]$$

The *C_p_* describes the bending degree of the focusing curve at the focal position. The greater the *C_p_*, the higher the sensitivity of the operator near the focal position. Similarly, if the step distance of the image acquisition system is unknown, *d* can be omitted.

### 2.5. Relative Root Mean Square Error (RRMSE)

**Figure 4:**
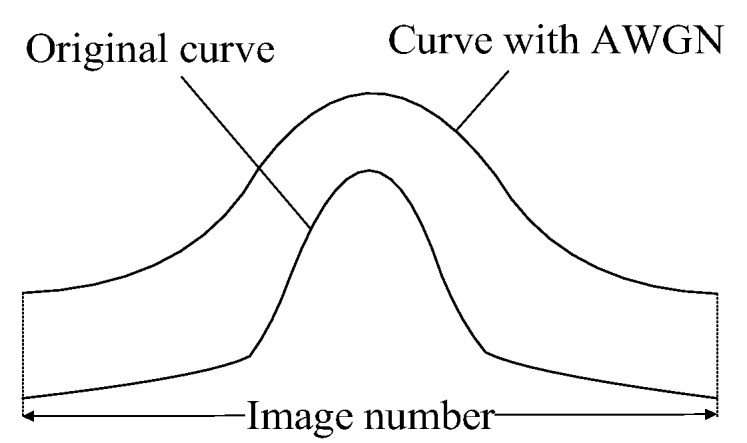
Comparison of curve with AWGN and original curve.

In focus measurement, variations in image noise (typically characterized by changes in noise variance) can cause fluctuations in the FMO’s output. The extent of these fluctuations indicates the FMO’s noise robustness. To assess the robustness of different FMOs, noise can be artificially introduced into the image, and the robustness of the operator can be evaluated by comparing the differences between the focus measure curves of the original image and the noise-added image. Additive White Gaussian Noise (AWGN) is a prevalent form of noise in imaging systems; therefore, adding it to images aligns with real-world conditions. Generally, the focusing function value of the image with AWGN is higher than that of the original image, as demonstrated in Figure 4.

A larger deviation between the original curve and the curve with AWGN indicates a reduced robustness of the focusing function. Therefore, this paper proposes using the relative root mean square error (*RRMSE*) to measure the robustness of the FMO. The *RRMSE* is defined in Equation (6).

(6) $$RRMSE=\frac{1}{M_{F}}\sqrt{\frac{1}{N}\sum_{k=1}^{N}{\left[F_{n}(k)-F(k)\right]}^{2}}$$

In Equation (6), *M_F_* denotes the arithmetic mean of all of the focus measure values calculated from the original images, where *F*(*k*) and *F_n_*(*k*) correspond to the focus measure values obtained from the original images and those corrupted by AWGN, respectively, with *N* indicating the total number of images.

Experimental results demonstrate that the *RRMSE* value is approximately proportional to the noise variance. In other words, the magnitude of noise variance does not affect the evaluation of FMOs by the *RRMSE* metric. Therefore, in practical applications, the noise variance can be selected based on specific requirements or the actual conditions of the image acquisition system.

Specifically, a smaller *RRMSE* indicates stronger resistance to noise interference, suggesting better robustness in practical applications. It should be noted that, due to the randomness of noise, the *RRMSE* may vary with each calculation, but the variation is generally small and negligible.

## 3. Focus Measure Operators

FMOs can be broadly categorized into four types: spatial domain-based focus measure operators, frequency domain-based operators, statistics-based operators, and information entropy-based operators [21]. Spatial domain-based FMOs are characterized by high sensitivity and low computational complexity. Statistics-based FMOs require minimal computation and offer good real-time performance. Frequency domain-based operators involve relatively higher computational complexity. These three types of operators are currently the most widely used FMOs. The information entropy-based operator uses entropy to measure the image sharpness. However, in most cases, the entropy difference between a sharp image and its blurred counterpart of the same scene is not significant. Moreover, noise (particularly AWGN) can significantly affect the image’s entropy value. Consequently, information entropy-based operators exhibit low sensitivity and poor noise robustness, making them unsuitable for practical applications.

In this paper, we select seven commonly used spatial domain-based focus measure operators, two statistics-based operators, and three frequency domain-based operators, and conduct a quantitative evaluation using the metrics proposed in this study.

(1) SMD

The Sum of Modified Differences (SMD) function evaluates the sharpness of an image by summing the absolute values of gray differences between adjacent pixels in the horizontal and vertical directions. Given an image size of *M* × *N*, the SMD operator can be formulated as Equation (7).

(7) $$F=\sum_{x=1}^{M-1}\sum_{y=1}^{N-1}\left[\left|I(x,y)-I(x+1,y)\right|+\left|I(x,y)-I(x,y+1)\right|\right]$$

(2) Roberts

The squared sum derived from the cross subtraction of the grayscale values of four adjacent pixels is adopted as the gradient value at each pixel in the Roberts operator. The gradient values of all pixels are then summed up to obtain the value of the measure function, as shown in Equation (8).

(8) $$F=\sum_{x=1}^{M-1}\sum_{y=1}^{N-1}\left\{{\left[I\left(x+1,y+1\right)-I\left(x,y\right)\right]}^{2}+{\left[I\left(x+1,y\right)-I\left(x,y+1\right)\right]}^{2}\right\}$$

(3) Tenengrad

The Tenengrad FMO can be viewed as a treatment of Sobel enhancement, which calculates the horizontal and vertical gradients of the image using the Sobel operator, which, in turn, measures the sharpness of the image edges. Equations (9) and (10) are the horizontal gradient *G_x_* and vertical gradient *G_y_* of the image obtained using the Sobel operator, respectively.

(9) $$G_{x}=\left(\begin{array}{ccc} 1 & 0 & -1\\ 2 & 0 & -2\\ 1 & 0 & -1 \end{array}\right)*I(x,y)$$

(10) $$G_{y}=\left(\begin{array}{ccc} 1 & 2 & 1\\ 0 & 0 & 0\\ -1 & -2 & -1 \end{array}\right)*I(x,y)$$

The * in Equations (9) and (10) represents the convolution symbol, and the Tenengrad FMO is shown in Equation (11).

(11) $$F=\sum_{x=2}^{M-1}\sum_{y=2}^{N-1}\left[G_{x}{\left(x,y\right)}^{2}+G_{y}{\left(x,y\right)}^{2}\right]$$

(4) Brenner

The Brenner FMO is a simple and effective method for image sharpness measurement, which obtains the focus measure value by squaring the difference in an adjacent 2-unit image pixel.

(12) $$F=\sum_{x=1}^{M-2}\sum_{y=1}^{N}{\left[I\left(x,y\right)-I\left(x+2,y\right)\right]}^{2}$$

(5) EOG

The Energy of Gradient (EOG) is employed as a FMO, where the evaluation score for each pixel is computed by summing the square of the differences in grayscale values between adjacent pixels along the *x* and *y* directions, as shown in Equation (13).

(13) $$F=\sum_{x=1}^{M-1}\sum_{y=1}^{N-1}\left\{{\left[I\left(x+1,y\right)-I\left(x,y\right)\right]}^{2}+{\left[I\left(x,y+1\right)-I\left(x,y\right)\right]}^{2}\right\}$$

(6) EOL

The Energy of Laplace (EOL) FMO is based on the Laplace operator, which is used as the FMO by calculating the square of the difference between the center point and the grayscale values in the four directions, as shown in Equation (14).

(14) $$F=\sum_{x=2}^{M-1}\sum_{y=2}^{N-1}{\left[I\left(x+1,y\right)+I\left(x-1,y\right)+I\left(x,y+1\right)+I\left(x,y-1\right)-4I\left(x,y\right)\right]}^{2}$$

(7) SML

The Sum of Modified Laplacian (SML) FMO is an improvement upon the EOL operator. It measures image sharpness by calculating the square of the sum of the absolute values of the second-order differences in the horizontal and vertical directions, as shown in Equation (15).

(15) $$F=\sum_{x=2}^{M-1}\sum_{y=2}^{N-1}{\left[\left|2I\left(x,y\right)-I\left(x-1,y\right)-I\left(x+1,y\right)\right|+\left|2I\left(x,y\right)-I\left(x,y-1\right)-I\left(x,y+1\right)\right|\right]}^{2}$$

(8) Variance

The Variance function is used to measure the degree of dispersion in the distribution of image gray values and is achieved by calculating the squared cumulative deviation of each pixel’s gray level from the mean value, as shown in Equation (16).

(16) $$F=\sum_{x=1}^{M}\sum_{y=1}^{N}{\left[I\left(x,y\right)-\bar{I}\right]}^{2}$$

In Equation (16), *Ī* represents the mean gray value of the image.

(17) $$\bar{I}=\frac{1}{MN}\sum_{x=1}^{M}\sum_{y=1}^{N}I\left(x,y\right)$$

(9) Vollath’s

The Vollath’s function evaluates image sharpness based on the correlation between neighboring pixels, as expressed in Equation (18).

(18) $$F=\sum_{x=1}^{M-2}\sum_{y=1}^{N}I\left(x,y\right)\left|I\left(x+1,y\right)-I\left(x+2,y\right)\right|$$

(10) Fourier transform based operator

The FMO based on Fourier transform is given by Equation (19).

(19) $$F_{FFT}=\sum_{u=-M/2}^{M/2-1}\sum_{v=-N/2}^{N/2-1}\sqrt{u^{2}+v^{2}}P\left(u,v\right)$$

In Equation (19) *P*(*u*,*v*) represents the power spectrum of the image and *P*(0,0) is the square of the DC component of the image.

(11) Discrete Cosine Transform-based operator

The FMO based on Discrete Cosine Transform (DCT) is given in Equation (20).

(20) $$F_{DCT}=\sum_{u=0}^{M-1}\sum_{v=0}^{N-1}\left(u+v\right)C^{2}\left(u,v\right)$$

In Equation (20), *C*(*u*,*v*) represents the DCT coefficient matrix, where *C*(0,0) corresponds to the DC component of the image.

(12) Wavelet Transform-based operator

Using the Daubechies6 (DB6) wavelet, the image is simultaneously decomposed through low-pass and high-pass filtering, resulting in four sub-images denoted as *w_LL_*, *w_LH_*, *w_HL_*, and *w_HH_*. Here, *w_LL_* represents the low-frequency components of the image, while *w_LH_* represents the components with low-frequency in the horizontal direction and the high-frequency in the vertical direction. Similarly, *w_HL_* corresponds to high-frequency horizontally and low-frequency vertically, and *w_HH_* contains the high-frequency components in the diagonal direction. Let *μ_LH_*, *μ_HL_*, and *μ_HH_* denote the mean values of the *w_LH_*, *w_HL_*, and *w_HH_* sub-images, respectively. The wavelet transform-based FMO is then expressed as in Equation (21).

(21) $$F_{DWT}=\sum_{\left(x,y\right)\in w_{LH}}{\left[w_{LH}\left(x,y\right)-\mu_{LH}\right]}^{2}+\sum_{\left(x,y\right)\in w_{HL}}{\left[w_{HL}\left(x,y\right)-\mu_{HL}\right]}^{2}+\sum_{\left(x,y\right)\in w_{HH}}{\left[w_{HH}\left(x,y\right)-\mu_{HH}\right]}^{2}$$

## 4. Experimental Analyses

### 4.1. Image Acquisitionc

**Figure 5:**
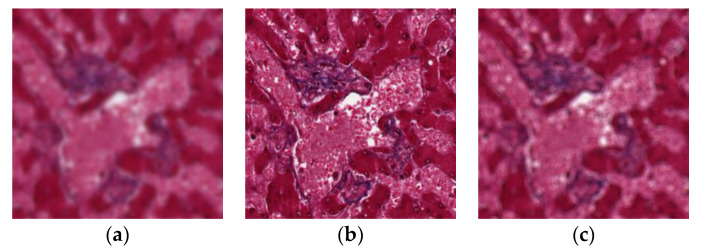
Pathological images: (**a**) 1st, (**b**) 9th, (**c**) 16th.

The proposed focusing evaluation metrics was initially validated using a publicly available dataset. This dataset comprises pathological images with a resolution of 1024 × 1024 pixels, totaling 16 images, with representative samples illustrated in Figure 5. The step distance for this dataset remains unspecified.

**Figure 6:**
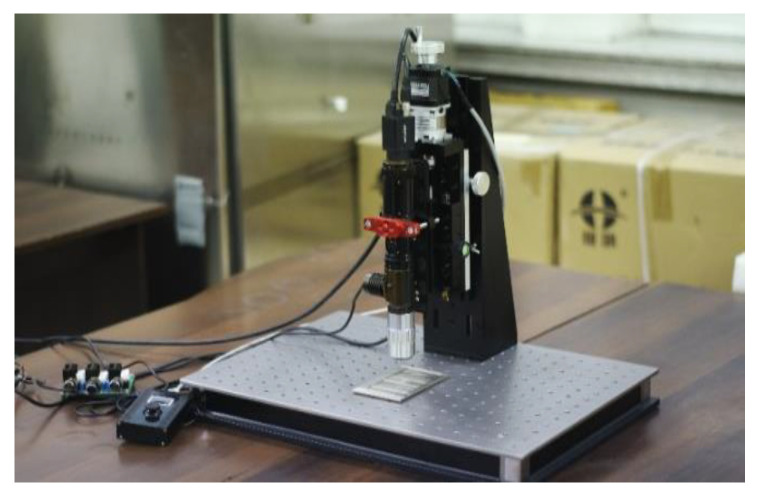
Focus variation imaging system.

This study constructed a microscopic imaging system, which adopts a reflective lighting mode, as shown in Figure 6. The camera used in the setup is the Hikvision (Hangzhou, China) MV-CA050-10 GM black-and-white camera. The objective lens uses Mitutoyo (Kawasaki City, Japan) M Plan APO 10×/0.28. The zoom lens tube provides magnification factors of 2× and 3.5×, respectively. The computer used in the setup runs on the Windows 10 Version1809 (64-bit) operating system, equipped with an Intel(R) Xeon E3-1220V3 @ 3.10 GHz processor, and the memory is 8 GB.

**Figure 7:**
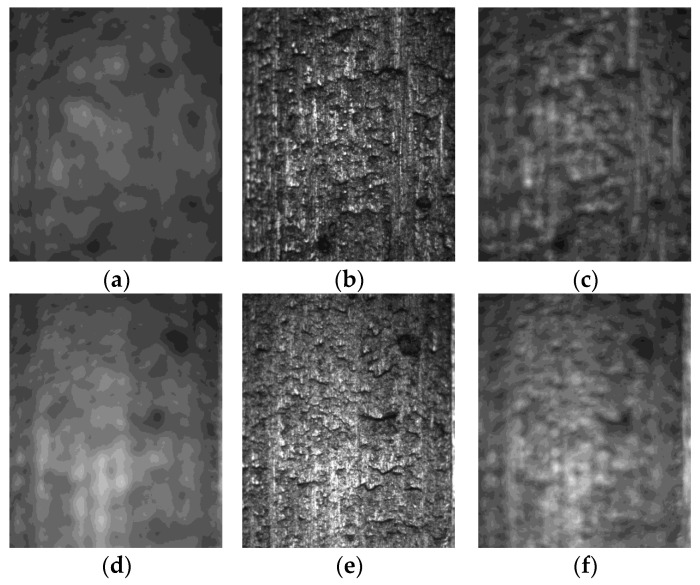

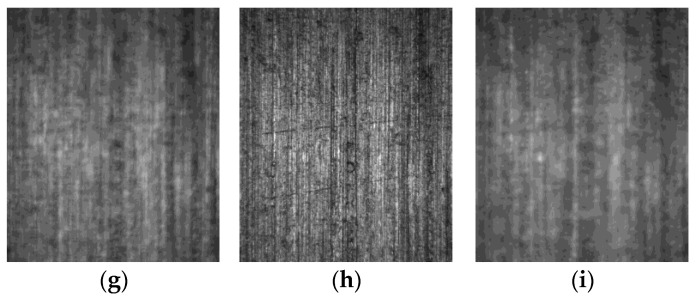
Planed surface images (step distance = 1.25 μm): (**a**) 1st, (**b**) 45th, (**c**) 90th; planed surface images (step distance = 2 μm): (**d**) 1st, (**e**) 40th, (**f**) 80th; ground surface images: (**g**) 1st, (**h**) 45th, (**i**) 90th.

In the industrial sector, planing and grinding are two common machining processes, and the surface textures produced by these processes show significant differences. The texture of the planed surfaces is more complex and irregular. In contrast, the texture of the ground surfaces appears as more regular micro-textures in images, with stronger directionality and a gentler overall surface. Utilizing these two types of machined surfaces helps to evaluate the performance of different algorithms when processing various surfaces. For the planed surface, when the zoom tube magnification is set to 2×, the sampling step distance is 2 μm and the number of sampled images is 80. When the zoom tube magnification is increased to 3.5×, the sampling step distance is reduced to 1.25 μm and the number of sampled images is 90. For the ground surface, the zoom tube magnification is fixed at 3.5×, with a sampling step distance of 1.25 μm and a total of 90 sampled images. The acquired images have a resolution of 1024 × 1224 pixels. Representative captured images are shown in Figure 7.

### 4.2. Analysis of Results

In order to facilitate the comparison of the performance of different focusing functions, it is generally necessary to normalize the function values. This study employs individual function normalization, in which each focusing function is divided by its maximum value.

**Figure 8:**
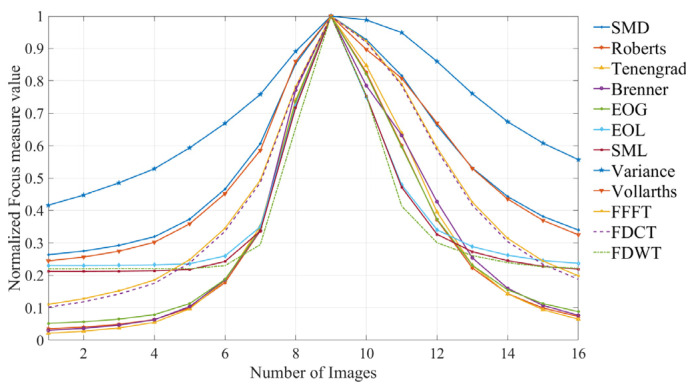
Focus measure curves from the pathological images.

The normalized focus measure curves derived from the pathological images using different FMOs are illustrated in Figure 8.

**Figure 9:**
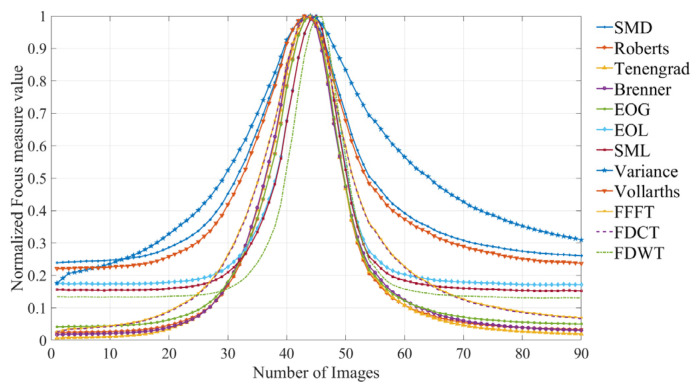
Focus measure curves from the planed surface images (step distance = 1.25 μm).

The normalized focus measure curves derived from the planed surface images (step distance = 1.25 μm) are illustrated in Figure 9.

**Figure 10:**
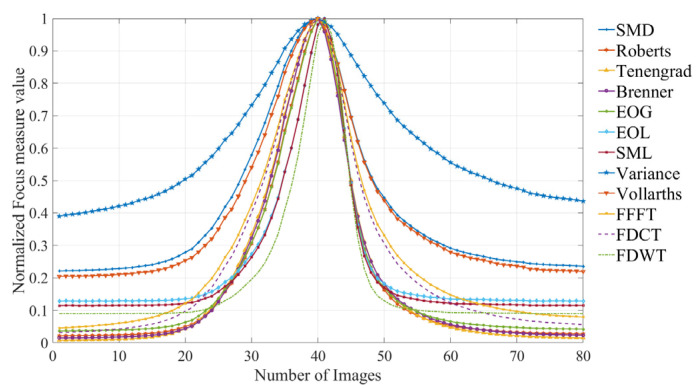
Focus measure curves from the planed surface images (step distance = 2 μm).

The normalized focus measure curves derived from the planed surface images (step distance = 2 μm) are illustrated in Figure 10.

**Figure 11:**
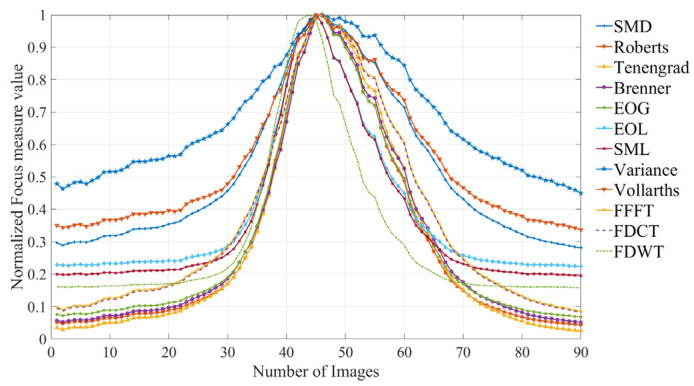
Focus measure curves from the ground surface images.

The normalized focusing curves derived from the ground surface images are illustrated in Figure 11.

To evaluate the performance of the proposed metrics, other indicators were introduced for comparison. The full width at half maximum (FWHM) is a commonly used method for measuring the width of waveform or signal amplitude distribution, which is compared to the *Ws* metric in this study. The peak slope (*Sp*) is a conventional indicator for assessing the sensitivity of FMOs near the peak point, as defined in Equation (22), and is compared with the *Cp* metric in this paper.

(22) $$S_{p}=\frac{2F(k_{max})-F(k_{max}+1)-F(k_{max}-1)}{2d}$$

Similarly, if the step distance of the image acquisition system is unknown, *d* can be omitted.

The evaluation metrics proposed in Section 2 were applied to analyze various FMOs. For pathological images, the fitting lines was constructed using three data points, with the slope utilized as the criterion for evaluating the fitted line in the gradual slope region.

For the planed surface, the fitted lines were constructed using seven data points at a step distance of 1.25 µm and five data points at 2 µm. For ground surfaces, seven data points were consistently employed for linear fitting. In both planed and ground surfaces, the RSS was adopted as the discrimination criterion for gradual slope region fitting.

To validate the *RRMSE* metric, AWGN with a variance of five was introduced to the images. Furthermore, to compare the computational efficiency of the operators, the average processing time per image was recorded for each operator.

The metrics from the pathological images are shown in Table 1. The numbers in parentheses indicate the performance ranking of the operators. In some exceptional cases (e.g., the variance operator in Table 1), FWHM may fail to return a valid result, which is then represented as NaN.

The metrics from the planed surface (step distance = 1.25 μm) are shown in Table 2.

The metrics from the planed surface (step distance = 2 μm) are shown in Table 3.

The metrics from the ground surface are shown in Table 4.

Experimental results indicate that, when the parameters of the image acquisition system or the acquired images change, the values of the *W_s_* metric exhibit significant variations, while the performance ranking of FMOs remains largely consistent. The FWHM metric shows similar behavior, but, in some exceptional cases, it may fail to produce results, whereas the *W_s_* metric consistently provides stable outputs. Thus, for evaluating the performance of operators, the *W_s_* metric demonstrates greater robustness.

Regarding the *R_sg_* metric, when the image acquisition system parameters or the acquired images change, the numerical values of *R_sg_* for most operators do not vary significantly, but their performance rankings undergo noticeable shifts. This is primarily because the *R_sg_* values of some operators are very close to each other.

For the *C_p_* metric, when the step distance of the image acquisition system increases, the *C_p_* value rises approximately proportionally, which aligns with theoretical expectations. When the acquired images change, the performance rankings of most operators based on *C_p_* remain stable, although some exceptions—such as the Vollath’s operator and wavelet-based operators—exhibit significant shifts. The Vollath’s operator extracts information from only one dimension of the image, while wavelet operators are sensitive to directional texture variations. Consequently, their performance varies considerably with changes in image content, necessitating caution in practical applications. The *S_p_* metric yields results similar to *C_p_*, but its values are roughly half those of *C_p_*, indicating that *C_p_* offers a wider dynamic range, facilitating better discrimination between operator performances. For example, in the case of a planed surface images with a step distance of 1.25 µm, the variation range of *C_p_* is 0.0400–0.0068 = 0.0332, whereas that of *S_p_* is only 0.0200–0.0034 = 0.0166.

For the *RRMSE* metric, increasing the step distance of the image acquisition system leads to a notable decrease in *RRMSE* values, although the operator performance rankings remain unchanged. The reduction in *RRMSE* is attributed to the decreased magnification, which enhances image brightness. Thus, increasing brightness improves the noise robustness of focus measure operators. When the acquired images change, the *RRMSE* values vary significantly, but the performance rankings remain stable. Notably, when transitioning from the planed surface to the ground surface, the *RRMSE* values of most operators decrease, whereas the *RRMSE* of the Brenner operator increases. The Brenner operator also extracts information from only one dimension of images, making its performance highly dependent on image content.

Among spatial-domain operators, the SMD operator demonstrates poor performance across all metrics. The Roberts, Tenengrad, Brenner, and EOG operators exhibit comparable performance in terms of *W_s_*, *R_sg_*, and *C_p_*, with the EOG operator generally outperforming the others in these three metrics. However, the EOG operator yields the worst *RRMSE* among the four, indicating its poor noise robustness. In contrast, the Tenengrad operator shows superior noise robustness.

The SML operator, an improved variant of the EOL, avoids the cancelation effect of positive and negative gradients by computing the absolute values of second-order differences along horizontal and vertical directions. Experimental results reveal that the EOL and SML operators perform similarly in *W_s_*, *R_sg_*, and *C_p_*, but the SML operator achieves a better *RRMSE*, suggesting enhanced noise robustness. Overall, both operators exhibit high sensitivity, but remain suboptimal in noise robustness.

Statistical-based operators, Variance and Vollath’s, demonstrate high computational efficiency. The Variance operator performs worst in sensitivity metrics, but best in *RRMSE*. However, when applied to SFF, replacing the global mean with local means significantly degrades its noise robustness. The Vollath’s operator shows no outstanding performance in either sensitivity or noise robustness.

Among the three frequency-domain operators, *F_FFT_* and *F_DCT_* exhibit similar performance in *W_s_*, *R_sg_*, and *C_p_*, with *F_DCT_* being slightly better. However, *F_FFT_* outperforms in *RRMSE* and computational efficiency. The *F_DWT_* operator excels in *W_s_*, *R_sg_*, and *C_p_*, but performs poorly in *RRMSE*.

In summary, frequency-domain operators exhibit significantly longer computation times, rendering them less practical for real-world applications. Among statistical operators, the Variance operator demonstrates extreme performance characteristics, while the Vollath’s operator shows no particularly outstanding features; both types, however, benefit from relatively short computation times. In the spatial domain, the EOL and SML operators achieve optimal sensitivity performance but demonstrate inferior noise robustness. The Roberts, Brenner, and Tenengrad operators maintain a favorable balance between sensitivity and noise robustness, with the Tenengrad operator exhibiting the most superior noise robustness among the three.

When the image acquisition system parameters or the acquired images change, the performance rankings of most operators remain stable. Based on an analysis of the algorithmic principles of various focus measure operators, the proposed metrics effectively distinguish their performance differences. In practical applications, researchers can select optimal operators by balancing sensitivity, noise robustness, and computational complexity according to specific scenario requirements. The proposed metrics provide a scientific basis for selecting FMOs in autofocus systems and SFF tasks.

## 5. Conclusions

This study investigates the quantitative evaluation of FMO performance from the morphological characteristics of a focus measure curve. The focus measure curve can be divided into steep and gradual slope regions, and accurately distinguishing these regions is critical. The proposed multi-point linear fitting method improves the objectivity and accuracy of curve segmentation compared to threshold-based approaches. Considering both accuracy and robustness, four metrics were designed: steep region width (*W_s_*), steep-to-gradual ratio (*R_sg_*), peak curvature (*C_p_*), and relative root mean square error (*RRMSE*). Experiments with commonly used FMOs demonstrate that when image acquisition parameters or image content change, the performance rankings of most operators remain stable, while significant variations in a few operators can be explained by their algorithmic principles. Thus, the proposed metrics robustly evaluate the performance of FMOs. Additionally, they provide theoretical guidance for designing new ones.

The proposed method requires the focus measure curve to exhibit a unimodal characteristic, implying that the measured object must approximate a flat plane. This constraint limits the applicability of the evaluation metrics. However, since the performance rankings of most operators are unaffected by changes in image content, a practical approach is to first select suitable FMOs using objects with planar features and then apply them to real objects. For the multi-point linear fitting method, increasing the number of fitting points enhances stability, which imposes requirements on the step distance and the number of acquired images. If the step distance is too large or the number of images is insufficient, the evaluation metrics may not meet application requirements. Additionally, determining the optimal number of fitting points requires further research, as current practices rely mainly on empirical judgment.

## Figures and Tables

**Table 1:** Metrics comparison of the pathological images.

	Ws	Rsg	Cp	RRMSE	FWHM	Sp	Time/ms
SMD	7.91 (10)	3.4821 (8)	0.2196 (11)	0.4870 (8)	7.11 (11)	0.1100 (11)	24.4 (4)
Roberts	6.71 (5)	4.6123 (7)	0.4374 (6)	0.3287 (6)	4.03 (4)	0.2194 (6)	25.0 (5)
Tenengrad	6.94 (6)	4.6608 (5)	0.4099 (7)	0.0726 (2)	4.19 (6)	0.2058 (7)	31.9 (7)
Brenner	7.26 (7)	5.0280 (4)	0.4432 (4)	0.2002 (5)	4.27 (7)	0.2216 (4)	23.5 (3)
EOG	6.65 (4)	4.6285 (6)	0.4416 (5)	0.6012 (9)	4.03 (4)	0.2214 (5)	26.8 (6)
EOL	5.58 (2)	7.6830 (2)	0.5273 (3)	2.9718 (11)	3.52 (3)	0.2637 (3)	33.8 (8)
SML	5.59 (3)	7.5626 (3)	0.5290 (2)	2.8498 (10)	3.47 (2)	0.2646 (2)	36.2 (9)
Variance	8.52 (12)	2.0959 (12)	0.1204 (12)	0.0045 (1)	NaN	0.0604 (12)	6.9 (1)
Vollath’s	7.94 (11)	3.4270 (9)	0.2428 (10)	0.4213 (7)	6.95 (10)	0.1215 (10)	16.8 (2)
*F_FF_* *_T_*	7.59 (9)	3.2517 (11)	0.2930 (9)	0.1047 (3)	5.54 (9)	0.1476 (9)	48.5 (10)
*F* *_DCT_*	7.58 (8)	3.2801 (10)	0.2992 (8)	0.1122 (4)	5.46 (8)	0.1507 (8)	88.1 (12)
*F* *_DWT_*	5.26 (1)	10.3261 (1)	0.5885 (1)	3.0205 (12)	3.18 (1)	0.2953 (1)	75.6 (11)

**Table 2:** Metrics comparison of the planed surface (step distance = 1.25 μm).

	Ws/mm	Rsg	Cp	RRMSE	FWHM/mm	Sp	Time/ms
SMD	0.0362 (11)	3.4708 (8)	0.0127 (11)	0.3956 (8)	0.0285 (11)	0.0064 (11)	16.1 (4)
Roberts	0.0280 (5)	4.2535 (5)	0.0244 (5)	0.2611 (6)	0.0159 (5)	0.0122 (5)	18.7 (6)
Tenengrad	0.0290 (6)	4.0672 (7)	0.0227 (7)	0.0651 (2)	0.0165 (6)	0.0113 (7)	28.3 (9)
Brenner	0.0291 (7)	4.1023 (6)	0.0246 (4)	0.1853 (5)	0.0167 (7)	0.0123 (4)	16.6 (5)
EOG	0.0278 (4)	4.3008 (4)	0.0242 (6)	0.4543 (9)	0.0160 (4)	0.0121 (6)	15.9 (3)
EOL	0.0238 (2)	4.9806 (3)	0.0391 (3)	1.4395 (12)	0.0153 (3)	0.0196 (3)	22.7 (8)
SML	0.0239 (3)	5.0140 (2)	0.0400 (1)	1.3602 (11)	0.0150 (2)	0.0200 (1)	21.6 (7)
Variance	0.0441 (12)	2.1603 (12)	0.0068 (12)	0.0082 (1)	0.0438 (12)	0.0034 (12)	5.7 (1)
Vollath’s	0.0346 (10)	3.3911 (9)	0.0151 (10)	0.3657 (7)	0.0266 (10)	0.0076 (10)	8.8 (2)
*F_FF_* *_T_*	0.0329 (9)	3.0643 (11)	0.0169 (9)	0.1346 (3)	0.0201 (9)	0.0085 (8)	60.7 (10)
*F* *_DCT_*	0.0328 (8)	3.0887 (10)	0.0170 (8)	0.1433 (4)	0.0199 (8)	0.0085 (8)	110.9 (12)
*F* *_DWT_*	0.0222 (1)	5.5932 (1)	0.0394 (2)	1.3072 (10)	0.0135 (1)	0.0197 (2)	88.9 (11)

**Table 3:** Metrics comparison of the planed surface (step distance = 2 μm).

	Ws/mm	Rsg	Cp	RRMSE	FWHM/mm	Sp	Time/ms
SMD	0.0508 (10)	3.2641 (10)	0.0148 (11)	0.2940 (8)	0.0402 (11)	0.0074 (11)	16.0 (4)
Roberts	0.0416 (5)	4.2818 (4)	0.0292 (6)	0.1726 (6)	0.0234 (5)	0.0146 (6)	18.0 (6)
Tenengrad	0.0439 (7)	4.1098 (7)	0.0265 (7)	0.0443 (2)	0.0247 (7)	0.0133 (7)	27.5 (9)
Brenner	0.0429 (6)	4.1555 (5)	0.0296 (4)	0.1159 (5)	0.0243 (6)	0.0148 (4)	15.1 (3)
EOG	0.0403 (4)	4.1287 (6)	0.0296 (4)	0.2998 (9)	0.0233 (4)	0.0148 (4)	16.3 (5)
EOL	0.0327 (2)	4.7539 (3)	0.0408 (3)	0.9165 (12)	0.0206 (3)	0.0204 (3)	21.5 (7)
SML	0.0330 (3)	4.7707 (2)	0.0412 (2)	0.8608 (11)	0.0205 (2)	0.0206 (2)	22.0 (8)
Variance	0.0688 (12)	2.6622 (12)	0.0077 (12)	0.0041 (1)	0.0929 (12)	0.0039 (12)	5.3 (1)
Vollath’s	0.0517 (11)	3.6621 (8)	0.0192 (10)	0.2674 (7)	0.0382 (10)	0.0096 (10)	8.7 (2)
*F_FF_* *_T_*	0.0494 (9)	3.2058 (11)	0.0207 (9)	0.0872 (3)	0.0302 (9)	0.0104 (9)	58.8 (10)
*F* *_DCT_*	0.0487 (8)	3.3196 (9)	0.0216 (8)	0.1020 (4)	0.0290 (8)	0.0108 (8)	106.8 (12)
*F* *_DWT_*	0.0293 (1)	5.0197 (1)	0.0487 (1)	0.7802 (10)	0.0173 (1)	0.0244 (1)	87.5 (11)

**Table 4:** Metrics comparison of the ground surface.

	Ws/mm	Rsg	Cp	RRMSE	FWHM/mm	Sp	Time/ms
SMD	0.0509 (11)	3.6154 (9)	0.0099 (11)	0.2475 (7)	0.0437 (10)	0.0049 (11)	15.1 (3)
Roberts	0.0446 (5)	4.9841 (4)	0.0197 (5)	0.1786 (5)	0.0276 (4)	0.0098 (5)	17.9 (6)
Tenengrad	0.0457 (7)	4.6457 (6)	0.0193 (6)	0.0450 (2)	0.0283 (6)	0.0097 (6)	26.4 (9)
Brenner	0.0456 (6)	4.4930 (7)	0.0200 (4)	0.1927 (6)	0.0284 (7)	0.0100 (4)	15.2 (4)
EOG	0.0445 (4)	5.0303 (3)	0.0192 (7)	0.3168 (9)	0.0281 (5)	0.0096 (7)	16.3 (5)
EOL	0.0378 (2)	5.2859 (1)	0.0305 (2)	1.0231 (12)	0.0270 (3)	0.0152 (2)	21.8 (8)
SML	0.0382 (3)	5.2614 (2)	0.0316 (1)	0.9484 (11)	0.0262 (2)	0.0158 (1)	21.2 (7)
Variance	0.0532 (12)	2.2964 (12)	0.0073 (12)	0.0075 (1)	0.0926 (12)	0.0037 (12)	6.4 (1)
Vollath’s	0.0496 (10)	3.8352 (8)	0.0243 (3)	0.3075 (8)	0.0459 (11)	0.0122 (3)	8.8 (2)
*F_FF_* *_T_*	0.0477 (9)	3.4663 (11)	0.0126 (10)	0.1155 (3)	0.0321 (9)	0.0078 (10)	57.6 (10)
*F* *_DCT_*	0.0476 (8)	3.4910 (10)	0.0160 (9)	0.1304 (4)	0.0319 (8)	0.0080 (9)	110.6 (12)
*F* *_DWT_*	0.0314 (1)	4.6550 (5)	0.0188 (8)	0.8909 (10)	0.0207 (1)	0.0094 (8)	88.3 (11)

## Data Availability

The data that support the findings of this study are available from the corresponding author upon reasonable request.
